# CDC Grand Rounds: Preventing Hospital-Associated Venous Thromboembolism

**Published:** 2014-03-07

**Authors:** Michael B. Streiff, P. Jeffrey Brady, Althea M. Grant, Scott D. Grosse, Betty Wong, Tanja Popovic

**Affiliations:** 1 Johns Hopkins Medical Institutions Anticoagulation Management Service; 2 Center for Quality Improvement and Patient Safety, Agency for Healthcare Research and Quality; 3 Div of Blood Disorders, National Center on Birth Defects and Developmental Disabilities, CDC; 4 Office of the Director, CDC

Deep venous thrombosis (DVT) is a blood clot in a large vein, usually in the leg or pelvis. Sometimes a DVT detaches from the site of formation and becomes mobile in the blood stream. If the circulating clot moves through the heart to the lungs it can block an artery supplying blood to the lungs. This condition is called pulmonary embolism. The disease process that includes DVT and/or pulmonary embolism is called venous thromboembolism (VTE). Each year in the United States, an estimated 350,000–900,000 persons develop incident VTE, of whom approximately 100,000 die, mostly as sudden deaths, the cause of which often goes unrecognized (1). In addition, 30%–50% of persons with lower-extremity DVT develop postthrombotic syndrome (a long-term complication that causes swelling, pain, discoloration, and, in severe cases, ulcers in the affected limb) (2,3). Finally, 10%–30% of persons who survive the first occurrence of VTE develop another VTE within 5 years (4).

VTE can result from three pathogenic mechanisms: hypercoagulability (increased tendency of blood to clot), stasis or slow blood flow, and vascular injury to blood vessel walls. Individual characteristics include congenital and acquired factors, such as advanced age or cancer, and interact with external factors, such as hospitalization or surgery (Table). Hospitalization is an important risk factor in the latter two mechanisms; injury and surgery are causes of vascular injury, and prolonged bed rest can cause stasis. Approximately half of new VTE cases occur during a hospital stay or within 90 days of an inpatient admission or surgical procedure, and many are not diagnosed until after discharge (5,6).

As a health-care–associated condition, VTE is receiving increased attention from patient safety experts, the Agency for Healthcare Research and Quality (AHRQ), and the Centers for Medicare & Medicaid Services (CMS). Despite that recognition, the number of secondary diagnoses of VTE in hospital patients has increased (7), and during 2007–2009, an average of nearly 550,000 adult hospital stays each year had a discharge diagnosis of VTE (8). Nonetheless, VTE often is not recognized as an issue of public health importance. No ongoing surveillance system monitors the occurrence of VTE at the population level, and public education and awareness is limited.

## Successful Implementation of a VTE Prophylaxis Program in the Inpatient Setting

Both pharmacologic and mechanical prophylaxis can be used to prevent VTE (9). Pharmacologic approaches, such as unfractionated and low molecular weight heparin and other anticoagulants (i.e., blood thinners), reduce the potential of blood to clot. Mechanical approaches such as intermittent pneumatic compression devices and graduated compression stockings can reduce blood clot formation by increasing blood flow. Patient adherence is essential for success with both approaches (10). Pharmacologic and mechanical methods of prophylaxis have different risks and benefits.

Prevention of VTE can be complicated because physicians must balance the risk for thrombosis with the risk for bleeding from anticoagulants by considering each patient’s risk for VTE and bleeding relative to the risks and benefits of prophylaxis. The 2012 American College of Chest Physicians VTE-prevention guidelines endorsed three quantitative risk-stratification models and suggested that VTE prophylaxis might not be beneficial for low-risk hospitalized patients (11–13). Assessment models for bleeding risk can be used to identify patients at high risk for bleeding (14). Because many cases of VTE are health-care associated, clinicians and health-care organizations can play an important role in preventing hospital-associated VTE (HA-VTE) events as part of patient-safety quality-improvement initiatives.

In 2004, the Johns Hopkins Medical Institutions Center for Innovations in Quality Patient Care assembled a multidisciplinary VTE-prevention team to develop a VTE education program for health-care providers, design evidence-based risk-appropriate prophylaxis strategies, establish a mechanism to assess performance, and review data with staff to improve performance (15,16). Paper-based order sets or forms were developed to guide clinicians through the risk-stratification process and recommend appropriate VTE prophylaxis. Among surgical patients, use of risk-appropriate VTE prophylaxis increased from 26% (42 of 161) at baseline to 68% (178 of 262) within 12 months. However, paper order sets were difficult for providers to use and made performance assessment labor-intensive. Therefore, computer-based “smart order sets” were designed and inserted as mandatory fields in all admission and transfer order sets for all surgical and medical patients. After this change, prescription of risk-appropriate VTE prophylaxis increased to approximately 85%, and all surgical and medical patients were risk-stratified for VTE (Figure) (15). A before/after study of outcomes for medical patients noted a 67% decrease in the frequency of confirmed symptomatic VTE within 90 days of hospital discharge, from 2.5% to 0.7%, and a 100% reduction in potentially preventable episodes of VTE (e.g., VTE that occurred with suboptimal VTE prophylaxis), from 1.1% to zero; no increase was observed in major bleeding events during hospitalization (16).

The Johns Hopkins experience emphasizes the key elements of an optimal VTE-prevention strategy: 1) VTE-prevention risk assessments must be a mandatory part of patient care; 2) clinicians must identify VTE risk factors and contraindications to prophylaxis; 3) clinicians must order risk-appropriate VTE prophylaxis; 4) patient risk factors must be reassessed during their hospital stay; 5) the system must collect patient and provider data to monitor performance; 6) adverse outcomes (e.g., hospital-acquired VTE and bleeding) must be monitored; and 7) performance must be measured regularly to promote continuous improvement (15).

## Prevention of VTE as an Overall Component of Patient Safety

VTE is the subject of numerous patient-safety quality or performance measures developed and promoted by federal agencies, such as AHRQ and CMS, and professional organizations, such as the Joint Commission and the National Quality Forum. Such measures are typically based on administrative data that are routinely collected and reported, but accurately ascertaining HA-VTE can be difficult without reviewing medical charts. In two studies conducted by the Institute for Healthcare Improvement using hospital patient chart reviews to identify adverse events, VTE was a fairly frequent cause of harm (eight events per 1,000 stays) and accounted for one out of 17 preventable deaths (17,18).

VTE is one of nine hospital-acquired conditions (HACs) targeted for an overall 40% reduction in preventable harms by the Partnership for Patients, a collaborative national health-care quality initiative led by CMS.* Hospital Engagement Networks are providing technical assistance to hospitals across the country to achieve the Partnership for Patients goals, including a reduction in 30-day readmissions.

AHRQ developed a VTE-prevention guide containing sample forms and protocols for clinicians to help implement processes to prevent VTE (19). It provides helpful resources and guides clinicians through key elements for change that need to be combined. Themes such as simplicity and ease of use are common to many successful quality-improvement efforts; clinicians are more likely to provide better care if it is easy to do so. AHRQ is in the process of revising the VTE-prevention guide to incorporate new information. AHRQ also has produced information guides for patients and consumers on how to prevent blood clots and dangers to be aware of when taking blood thinners.†

Patient safety improvements can help achieve the “triple aim” as defined by the Institute for Healthcare Improvement: 1) to provide better patient experience of health care, 2) to improve population health, and 3) to decrease health-care costs (20). A positive patient safety culture fosters mutual trust, openness, and shared institutional goals (21). Efforts to prevent VTE share many of the same opportunities observed in patient safety in general. Like all patient safety initiatives, VTE prevention relies on a culture that is conducive to patient safety. There are compelling examples of institutions, in addition to Johns Hopkins, that have driven rates of VTE down to low levels, and some of them are helping others to achieve similar success (22). A collaborative, team-based approach to care is not only required for significant and sustained improvement, it also offers efficiency and capacity to tackle other patient safety problems (23).

## Public Health Strategies to Prevent HA-VTE

In 2011, CDC convened an expert panel to discuss prevention of HA-VTE. The experts identified the need for strategies to address the use of VTE prophylaxis among hospital patients and better ways to track HA-VTE.§ A recent publication summarized current HA-VTE prevention guidelines and evaluated risk assessment models (13). Multiple tools and approaches to assessing patient risk for HA-VTE have been proposed and implemented, but there is a lack of research to validate these tools and to identify which ones can best identify patients who should receive VTE prophylaxis (and if so, what type of prophylaxis). Comparative effectiveness research to quantify the relative performance of risk assessment models for VTE and bleeding is urgently needed.

Surveillance will be critical for assessing the impact of interventions to reduce HA-VTE. A comprehensive surveillance approach would collect information not only on the incidence of VTE but also information on the prevention practices implemented to assess the relationship between them. However, there are multiple major challenges for conducting surveillance for VTE. First, for various reasons, diagnosis codes for VTE in administrative databases often do not accurately identify patients with acute VTE. One strategy to minimize false positives for VTE in outpatient records is to require confirmation of a diagnosis code through the appearance of the same code in subsequent encounters and a filled prescription of an anticoagulant. However, because of false positives, only review of medical records in which the results of imaging tests document VTE can validate a diagnosis. Second, distinguishing new from recurrent VTE is challenging because an accurate medical history is needed but often is not available from administrative data sources. Third, mortality attributable to pulmonary embolism can lead to missed cases because of sudden death; thus, collecting additional information from autopsies and death records is critical for capturing cases and outcomes. Fourth, because VTE can be asymptomatic as well as symptomatic, temporal trends in VTE incidence might reflect institutional variations in screening and diagnosis practices instead of actual changes in overall incidence. Information on why screening was done is important for distinguishing these situations. Finally, because many cases of HA-VTE occur after discharge, data must be collected from multiple settings in which VTE is diagnosed and treated. Therefore, in 2012, CDC funded two pilot surveillance programs for a 2-year project to develop methods that combine use of administrative data with review of electronic medical records to yield more complete population-based estimates of VTE incidence and to inform the development of surveillance methods to overcome the challenges described. Data and methods from these pilot surveillance programs will lay the foundation for more accurate ongoing monitoring of VTE nationally.

## Conclusion

VTE is a problem of major public health importance, with hundreds of thousands of persons affected each year. Because nearly half of VTE cases occur during or soon after a hospital stay, there is overlap between VTE as a public health problem and a preventable patient safety problem. Public health programs and patient safety stakeholders, such as hospital networks and health-care payers, are encouraged to collaborate to promote effective risk-stratification and VTE prevention in inpatient settings and to assess trends in the use of risk-appropriate VTE prophylaxis for HA-VTE events and complications.

## Figures and Tables

**FIGURE:**
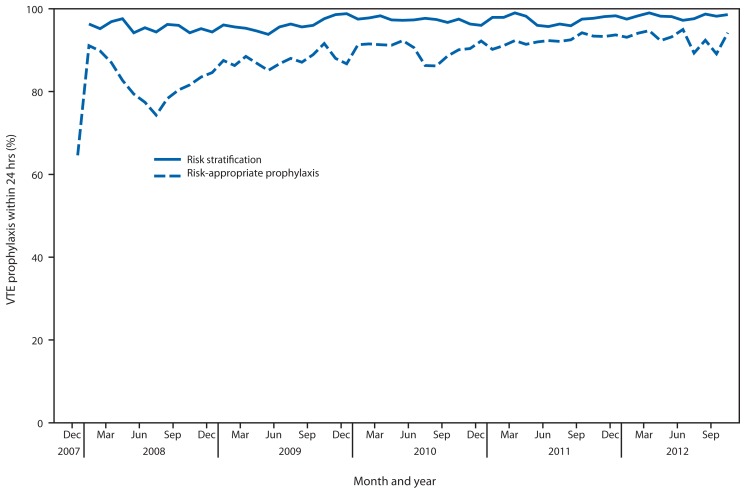
Venous thromboembolism (VTE) risk stratification and percentage of patients for whom risk-appropriate VTE prophylaxis was prescribed within 24 hours of hospital admission — Johns Hopkins Medical Institutions, 2008–2012

**TABLE:** Risk factors for venous thromboembolism (VTE)

Strong risk factors	Moderate risk factors	Weak risk factors
Fracture (hip or leg)	Arthroscopic knee surgery	Prolonged bed rest
Hip or knee replacement	Central venous lines	Immobility
Major general surgery	Chemotherapy/Cancer	Age >40 yrs
Major trauma	Congestive heart or respiratory failure	Laparoscopic surgery
Spinal cord injury	Estrogen	Obesity
	Age >65 yrs	Pregnancy
	Paralytic stroke	Varicose veins
	Postpartum period	
	Previous VTE	
	Thrombophilia	

**Source:** Anderson FA Jr, Spencer FA. Risk factors for venous thromboembolism. Circulation 2003;107(23 Suppl 1):I9–16.

## Footnotes

* Additional information available at http://www.ahrq.gov/research/findings/nhqrdr/nhqr10/chap3.html#support.

† Available at http://www.ahrq.gov/patients-consumers/diagnosis-treatment/treatments/btpills/index.html.

§ Additional information available at http://www.cdc.gov/ncbddd/dvt/documents/12_232434-a_sayers_ha-vte_workshop_report_508.pdf.

## References

[1] US Department of Health and Human Services (2008). The Surgeon General’s call to action to prevent deep vein thrombosis and pulmonary embolism.

[2] Kahn SR, Shrier I, Julian JA (2008). Determinants and time course of the postthrombotic syndrome after acute deep venous thrombosis. Ann Intern Med.

[3] Prandoni P, Kahn SR (2009). Post-thrombotic syndrome: prevalence, prognostication and need for progress. Br J Haematol.

[4] Kyrle PA, Rosendaal FR, Eichinger S (2010). Risk assessment for recurrent venous thrombosis. Lancet.

[5] Heit JA, Silverstein MD, Mohr DN (2001). The epidemiology of venous thromboembolism in the community. Thromb Haemost.

[6] Spencer FA, Emery C, Joffe SW (2009). Incidence rates, clinical profile, and outcomes of patients with venous thromboembolism: the Worcester VTE study. J Thromb Thrombolysis.

[7] Stein PD, Matta F, Dalen JE (2011). Is the campaign to prevent VTE in hospitalized patients working?. Chest.

[8] CDC (2012). Venous thromboembolism in adult hospitalizations—United States, 2007–2009. MMWR.

[9] Kahn SR, Morrison DR, Cohen JM (2013). Interventions for implementation of thromboprophylaxis in hospitalized medical and surgical patients at risk for venous thromboembolism. Cochrane Database Syst Rev.

[10] Shermock KM, Lau BD, Haut ER (2013). Patterns of non-administration of ordered doses of venous thromboembolism prophylaxis: implications for novel intervention strategies. PLoS ONE.

[11] Gould MK, Garcia DA, Wren SM (2012). Prevention of VTE in nonorthopedic surgical patients: antithrombotic therapy and prevention of thrombosis. Chest.

[12] Kahn SR, Lim W, Dunn AS (2012). Prevention of VTE in nonsurgical patients: antithrombotic therapy and prevention of thrombosis. Chest.

[13] Maynard G, Jenkins IH, Merli GJ (2013). Venous thromboembolism prevention guidelines for medical inpatients: mind the (implementation) gap. J Hosp Med.

[14] Decousus H, Tapson VF, Bergmann JF (2011). Factors at admission associated with bleeding risk in medical patients: findings from the IMPROVE investigators. Chest.

[15] Streiff MB, Carolan HT, Hobson DB (2012). Lessons from the Johns Hopkins Multi-Disciplinary Venous Thromboembolism (VTE) Prevention Collaborative. BMJ.

[16] Zeidan AM, Streiff MB, Lau BD (2013). Impact of a venous thromboembolism (VTE) prophylaxis “smart order set”: improved compliance, fewer events. Am J Hematol.

[17] Classen DC, Resar R, Griffin F (2011). ‘Global trigger tool’ shows that adverse events in hospitals may be ten times greater than previously measured. Health Aff (Millwood).

[18] Landrigan CP, Parry GJ, Bones CB, Hackbarth AD, Goldmann DA, Sharek PJ (2010). Temporal trends in rates of patient harm resulting from medical care. N Engl J Med.

[19] Maynard G, Stein J (2008). Preventing hospital-acquired venous thromboembolism: a guide for effective quality improvement.

[20] Berwick DM, Nolan TW, Whittington J (2008). The triple aim: care, health, and cost. Health Aff (Millwood).

[21] Sorra JS, Dyer N (2010). Multilevel psychometric properties of the AHRQ hospital survey on patient safety culture. BMC Health Serv Res.

[22] Maynard G, Stein J (2010). Designing and implementing effective venous thromboembolism prevention protocols: lessons from collaborative efforts. J Thromb Thrombolysis.

[23] Clancy CM, Tornberg DN (2007). TeamSTEPPS: assuring optimal teamwork in clinical settings. Am J Med Qual.

